# The dynamic monitoring of aeolian desertification land distribution and its response to climate change in northern China

**DOI:** 10.1038/srep39563

**Published:** 2016-12-22

**Authors:** Lili Feng, Zhiqing Jia, Qingxue Li

**Affiliations:** 1 Institute of Desertification Studies, Qinghai Gonghe Desert Ecosystem Research Station, Chinese Academy of Forestry, Beijing, 100091, China

## Abstract

Aeolian desertification is poorly understood despite its importance for indicating environment change. Here we exploit Gaofen-1(GF-1) and Moderate Resolution Imaging Spectroradiometer (MODIS) data to develop a quick and efficient method for large scale aeolian desertification dynamic monitoring in northern China. This method, which is based on Normalized Difference Desertification Index (NDDI) calculated by band1 & band2 of MODIS reflectance data (MODIS09A1). Then we analyze spatial-temporal change of aeolian desertification area and detect its possible influencing factors, such as precipitation, temperature, wind speed and population by Convergent Cross Mapping (CCM) model. It suggests that aeolian desertification area with population indicates feedback (bi-directional causality) between the two variables (P < 0.05), but forcing of aeolian desertification area by population is weak. Meanwhile, we find aeolian desertification area is significantly affected by temperature, as expected. However, there is no obvious forcing for the aeolian desertification area and precipitation. Aeolian desertification area with wind speed indicates feedback (bi-directional causality) between the two variables with significant signal (P < 0.01). We infer that aeolian desertification is greatly affected by natural factors compared with anthropogenic factors. For the desertification in China, we are greatly convinced that desertification prevention is better than control.

Desertification is a type of land degradation in which a relatively dry land region becomes increasingly arid, typically losing its bodies of water as well as vegetation and wildlife. It is caused by a variety of factors, such as climate change and human activities^1^. Meanwhile aeolian desertification is the most important desertification type in China with serious environmental and socioeconomic problems in arid, semi-arid, and dry sub- humid zones. Continuous aeolian desertification has a serious influence on the biosphere. It is also highly related to issues such as declining productivity, biodiversity loss, land degradation, and declining ecosystem services^2^,^3^,^4^,^5^,^6^. Many studies showed that desertification was resulting from various processes and reasons including natural and anthropogenic factors^7^,^8^,^9^,^10^,^11^. Here we used Convergent Cross Mapping (CCM) model to explore the causality of aeolian desertification. The result shows that natural factors are the primary reason for aeolian desertification in northern China during the past 15 years.

In China, desertification area survey was conducted once every five years since 1994. Desertification lands occupy an area about 2.61 million km^2^ and spread across 18 provinces accounting for 27.20% of the country’s land area by 2014^12^. This survey takes a lot of manpower, material and financial resources with a lack of sequential dynamic monitoring. In this study, a remote sensing method was used to obtain the aeolian desertification land distribution in northern China. A new spectral index called Normalized Difference Desertification Index (NDDI) derived from MODIS surface reflectance data was used to acquire the aeolian desertification land distribution. Results will provide a basis for combating desertification. In northern China, natural vegetation is being transformed into agricultural lands at a faster rate, endangering ecosystem services and increasing soil-loss potential, which may trigger land degradation. This region is sensitive to climate change and human intervention. It becomes an original region of sandstorms. To alleviate the multifaceted environmental degradation, Chinese government has implemented several ecological restoration programs that have deeply affected the structure and function of grassland ecosystems. Understanding desertification processes and causes are important to provide reasonable and effective control measures for preventing desertification. The study area of this paper is located in northern China (31°09′N- 53°23′N, 73°40′E- 126°04′E) (Fig. 1). It includes Xinjiang Uyghur Autonomous Region, Qinghai Province, Gansu Province, Ningxia Hui Autonomous Region and Inner Mongolia Autonomous Region with eight famous deserts in China. Generally, a semi-arid or desert climate prevails in Xinjiang. The entire region is marked by great seasonal differences in temperature. This region includes Gurban Tunggut Desert, Taklamakan Desert and Kumtag Desert. Qinghai has quite cold winters, mild summers, and a large diurnal temperature variation. Significant rainfall occurs mainly in summer, while precipitation is very low in winter and spring, and is generally low enough to keep much of the province semi-arid or arid. This region includes Qaidam Basin Desert. Gansu generally has a semi-arid to arid continental climate with warm to hot summers and cold to very cold winters. Most of the limited precipitation is delivered in the summer months. This region includes Badain Jaran Desert, Tengger Desert and Kumtag Desert. Ningxia Hui Autonomous Region has a continental climate with average summer temperatures rising to 17 to 24 °C in July and average winter temperatures dropping to between −7 to −15 °C in January. Annual rainfall averages from 190 to 700 millimetres, with more rain falling in the south of the region. This region includes Tengger Desert in Shapotou. Inner Mongolia has a wide variety of regional climates. The winters in Inner Mongolia are very long, cold and dry. The spring is short, mild and arid, with large, dangerous sandstorms, whilst the summer is very warm to hot and relatively humid except in the west where it remains dry. Autumn is brief and sees a steady cooling, with temperatures below 0 °C reached in October in the north and November in the south. It includes Badain Jaran Desert, Tengger Desert, Kubuqi Desert and Ulan Buh Desert.

## Results

### Automatic monitoring of aeolian desertification land

**Figure 2:**
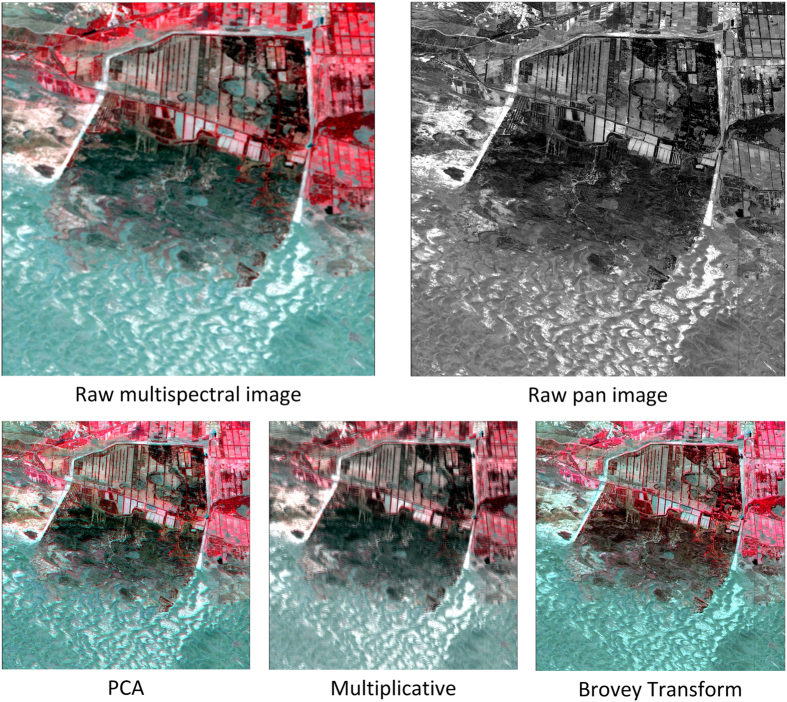
Image fusion results of GF-1 data based on three commonly used algorithms generated from ArcGIS 9.3 software developed by ESRI (Environmental Systems Research Institute) and ERDAS IMAGINE 9.1 software developed by Leica Geosystems Geospatial Imaging, LLC. ArcGIS 9.3 software was downloaded from http://arcmap.software.informer.com/9.3/. ERDAS IMAGINE 9.1 software was downloaded from http://erdas-imagine.software.informer.com/9.1/. The result shows that Principal Component Analysis (PCA) image fusion algorithm is the best choice for GF-1 data.

**Figure 3:**
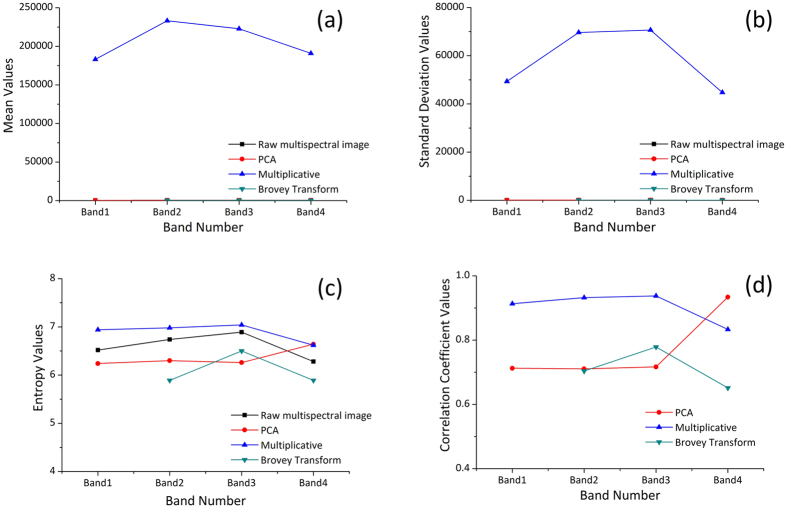
Parameter values change for different fusion algorithms. Mean and standard deviation values of fusion image using multiplicative algorithm are far from raw image. Entropy and correlation coefficient values of fusion image using PCA algorithm are greater than Brovey Transform.

Taking a part of GF-1 data as the experimental data (Fig. 2), three fusion algorithms as commonly used in the image fusion experiments were applied. Mean and standard deviation values of fusion image using multiplicative algorithm are far from raw image (Fig. 3a,b). Entropy and correlation coefficient values of fusion image using PCA algorithm are greater than Brovey Transform (Fig. 3c,d). It shows that Principal Component Analysis (PCA) image fusion algorithm is the best choice for GF-1 data fusion (Table 1). Then PCA image fusion algorithm was finally used on entire GF-1 data.

**Figure 4:**
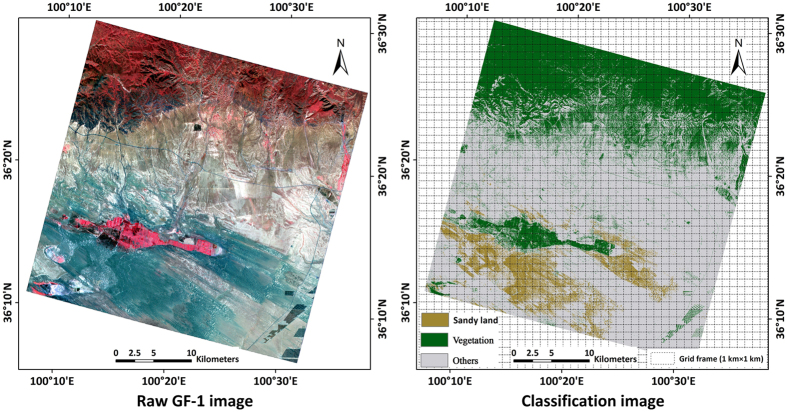
Boundary of GF-1 data and classification result generated from ArcGIS 9.3 software developed by ESRI (Environmental Systems Research Institute). ArcGIS 9.3 software was downloaded from http://arcmap.software.informer.com/9.3/. The raw GF-1 image is shown on the left; classification image is shown on the right.

GF-1 remote sensing image was classified by Support Vector Machine (SVM) classification algorithm. The main land cover type obtained by this algorithm is shown in Fig. 4. Results show that the SVM classification algorithm can meet the precision requirements based on visual interpretation of GF-1.

It is a problem to achieve the combination of high and low resolution remote sensing data. In this study, the first step was to establish a 1 km × 1 km grid frame (consistent with the resolution of re-sampled MODIS) with vector format and each grid was identified with a unique identity (Fig. 4). The second step was to use this frame to respectively perform statistical analysis for SVM classification result by GF-1 data in each grid. Finally, calculate the proportion of each land use type in each grid frame. Taking the proportion of aeolian desertification area was greater than 70% as the pure pixel for aeolian desertification land, the proportion of vegetation (others) area was greater than 90% as the pure pixel for vegetation (others). Then changes of reflectance values for different land use types were acquired. Total correlation index(r) value of all types between band1 and band2 was the lowest. Band1 and band2 exhibited a large disparity in their spectral responses of different land covers. So these two bands were used to derive Normalized Difference Desertification Index (NDDI) in this study.

**Figure 5:**
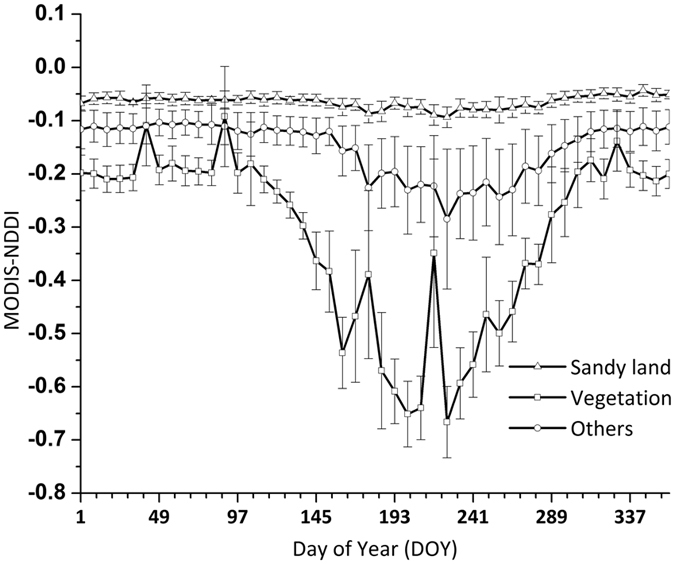
MODIS-NDDI time series curves of different land use types. These time series curves are based on the classification result of GF-1 data and time series curves of MODIS- NDDI.

**Figure 6:**
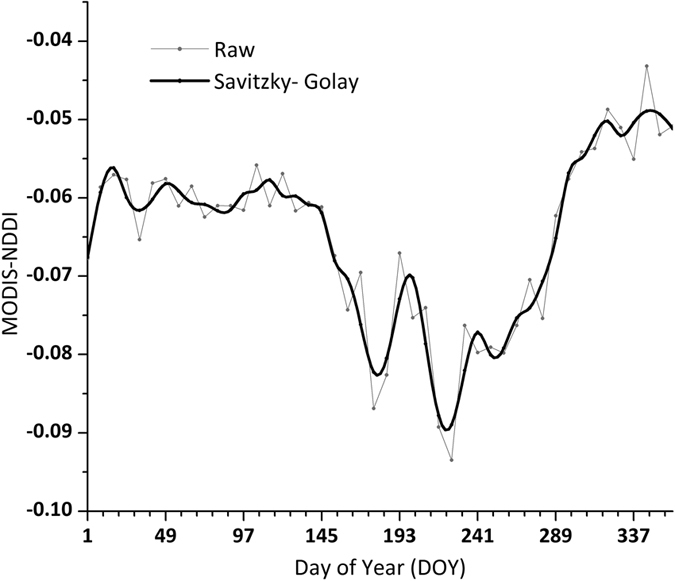
MODIS-NDDI time series curves of aeolian desertification land. Thin line is the raw MODIS-NDDI time series curve of aeolian desertification land; thick line is the filtered time series curve of aeolian desertification land using Savitzky-Golay algorithm to reduce noise.

Aeolian desertification area was extracted through above-mentioned method by using GF-1data. Mean value of MODIS-NDDI time series curves for different land use types are shown in Fig. 5. Filtered MODIS-NDDI time series curve of aeolian desertification land is shown in Fig. 6.

**Figure 7:**
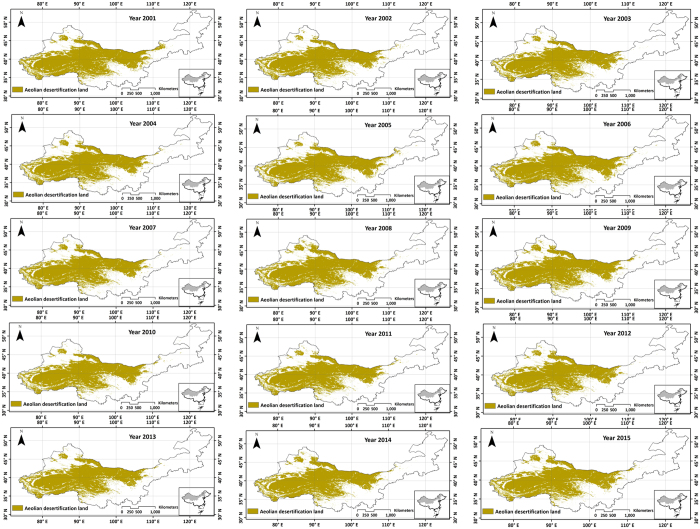
Aeolian desertification land distribution in northern China generated from ArcGIS 9.3 software developed by ESRI (Environmental Systems Research Institute) and ERDAS IMAGINE 9.1 software developed by Leica Geosystems Geospatial Imaging, LLC. ArcGIS 9.3 software was downloaded from http://arcmap.software.informer.com/9.3/. ERDAS IMAGINE 9.1 software was downloaded from http://erdas-imagine.software.informer.com/9.1/. Yellow parts are the aeolian desertification land distribution regions.

Mean Absolute Distance (MAD)^13^ was used to compare the MODIS-NDDI time series image of aeolian desertification land with the MODIS-NDDI image of the study area for each pixel. A lower image value illustrated a closer MAD, which indicated a greater possibility of aeolian desertification. A threshold was set on the MAD map based on the prior knowledge, by considering the official data of aeolian desertification area. The difference between the estimated aeolian desertification area and the official data was smallest when the threshold value is 0.051. In this study, a p-tile algorithm was adopted for threshold selection^14^. Aeolian desertification distribution of northern China in 2001 to 2015 is shown in Fig. 7. The eolian desertification land area estimated from MODIS and investigation data are shown Table 2.

### Factor analysis

**Figure 8:**
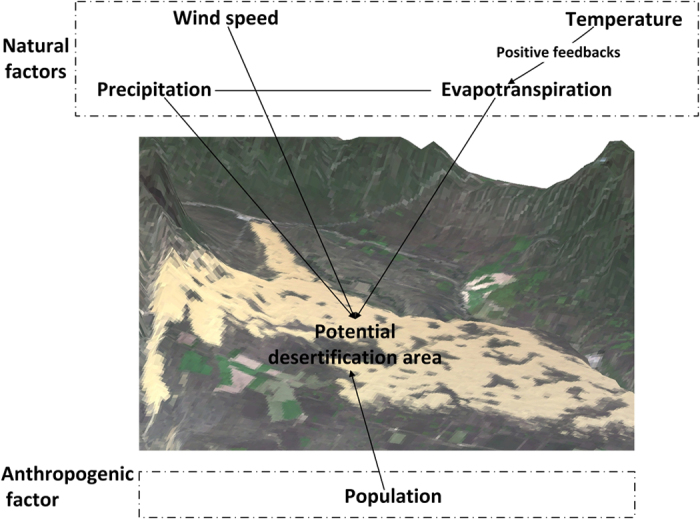
Possible influencing factors for desertification in northern China. Three-dimensional image generated from ArcGIS 9.3 software developed by ESRI (Environmental Systems Research Institute). ArcGIS 9.3 software was downloaded from http://arcmap.software.informer.com/9.3/. Desertification means the ratio of annual precipitation to potential evapotranspiration falls within the range from 0.05 to 0.65; and evapotranspiration is highly related to temperature. Wind is the power of desertification. Meanwhile, population is one of the most important anthropogenic factors of desertification.

Generally, desertification means the ratio of annual precipitation to potential evapotranspiration falls within the range from 0.05 to 0.65; and evapotranspiration is highly related to temperature. Wind is the power of desertification. Meanwhile, population is one of the most important anthropogenic factors of desertification. So temperature, precipitation and wind speed as the natural factors and population as the anthropogenic factor in combination of CCM model were used to analyze cause-and-effect relationship in this study (Fig. 8).

**Figure 9:**
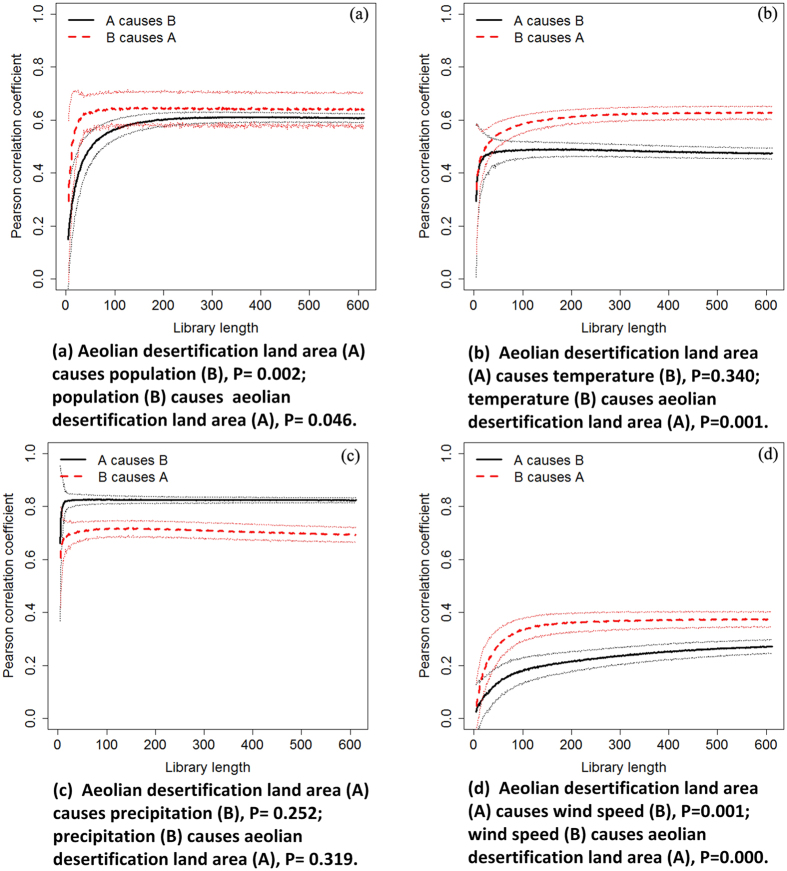
Predictive-skill curves based on Pearson correlation coefficients for convergent cross-mapping of aeolian desertification land area with the possible influencing factors. Dotted lines on either side of the predictive-skill curves represent the ±standard deviation of estimate assessed from bootstrapping based on 1000 iterations. Convergent cross-mapping is based on procedures written in the R-programming language initially developed by Clark *et al*.^25^. Pearson correlation coefficient represents the correlation between two variables. Library length is the number of historical observations, including observation time and number of spatial replicates included in the composite time series^24^,^25^.

Based solely on the relationship between library length and Pearson correlation coefficient, results for this CCM test suggest that aeolian desertification area with population indicates feedback (bi-directional causality) between the two variables (P < 0.05; Fig. 9a), but forcing of aeolian desertification area by population is weak. Based on the same diagnostic tests as we used above, we find aeolian desertification area is significantly affected by temperature, as expected (Fig. 9b). However, there is no obvious forcing for the aeolian desertification area and precipitation (Fig. 9c). Aeolian desertification area with wind speed indicates feedback (bi-directional causality) between the two variables with significant signal (P < 0.01; Fig. 9d).

## Discussion

Land degradation and desertification has been ranked as a major environmental and social issue in the coming decades in China. It has received a great attention, especially the northern China. Desertification area in China has increased since the 1950s and reached its maximum during the 1970s and early 1980s. Since then desertification area has decreased continuously to the present^15^,^16^,^17^. It suggests that reforestation/afforestation policy has played a significant role in controlling the desertification in China. Small field experiments prove that vegetation in desertified/degraded land could recover if isolated from human activities. Since 1998, natural recovery has become one powerful national force to prevent land desertification and recover natural vegetation^17^, so anthropogenic factors are not the main factor exacerbating the desertification distribution during 2001 to 2015 in northern China. Numerous scientists have claimed that land desertification in China is primarily due to human impacts. Wang *et al*.^15^ suggested that desertification in China has been primarily caused by climate change^15^. We also believed that desertification in northern China is mainly controlled by natural factors during the past 15 years. In addition, although overall land desertification area decreases year by year, desertification situation in some regions shows the worsened tendency, such as regions around rivers and lakes. We must pay more attention to environment deterioration of rivers, lakes and the nearby areas in the future. In this study, the proposed NDDI is able to obtain the aeolian desertification land distribution on a large scale and assess the aeolian desertification area variability simply and effectively. The results show that aeolian desertification area with population indicates feedback (bi-directional causality) between the two variables (P < 0.05). Forcing of aeolian desertification area by population is weak (P = 0.046). However, aeolian desertification area forcing population has more significant signal (P = 0.02). In fact, land desertification in northern China is affected by anthropogenic factors with small fluctuation. Once the land desertification happens, it will bring population migration with population growth pressure around desertification regions. Based on the same diagnostic tests as we used above, we find aeolian desertification area is significantly affected by temperature, as expected. However, there is no obvious forcing for the aeolian desertification area and precipitation. We supposed several situations about this: (i) there is a small fluctuation for the precipitation in northern China during the past 15 years, so we derived no obvious causality; (ii) precipitation is not the root cause of land desertification. Aeolian desertification area with wind speed indicates feedback (bi-directional causality) between the two variables with significant signal (P < 0.01). In conclusion, we infer that aeolian desertification is greatly affected by natural factors compared with anthropogenic factors. From the result, aeolian desertification area covers a large area in northern China threatening human life. If the desertification land area continues to grow, it will reduce the habitable zone and lead to a large number of population migration and growth^18^. It is also a big dust origin in northern China. For the desertification in China, we are greatly convinced that desertification prevention is better than control. We should pay more attention to the impact of climate change on the desertification distribution. Further, there are still many challenges in estimation of eolian desertification land area. The simulation time only chooses 15 years (2001–2015), and limited impact factors including precipitation, temperature, wind speed and population were used to analyze the cause-and-effect relationship. More field survey data in combination of more high spatial resolution images should also be adopted in the further research.

## Methods

In this study, the main processes to effectively estimate aeolian desertification land distribution include: (1) two aeolian desertification sensitive bands (band1 and band2) of MODIS09A1 were selected to calculate Normalized Difference Desertification Index (NDDI); (2) standard image of aeolian desertification land was generated by mean MODIS-NDDI time series curve of aeolian desertification land; (3) Mean Absolute Distance(MAD) was calculated between the MODIS-NDDI time series image and the standard image of aeolian desertification land; (4) aeolian desertification distribution from 2001 to 2015 was obtained; then spatial-temporal change of aeolian desertification area and its possible influencing factors were analyzed, such as precipitation, temperature, wind speed and population by Convergent Cross Mapping (CCM) model.

### MOD09A1 data

MOD09A1 data were downloaded for tiles h25v03, h26v03, h23v04, h24v04, h25v04, h26v04, h27v04, h23v05, h24v05, h25v05, h26v05 (h for horizontal, v for vertical) from 2001 to 2015 from the National Aeronautics and Space Administration (NASA) (http://earthdata.nasa.gov/). It provides eight days composite with a 500 m spatial resolution data. The red bands such as band1 (620–670 nm), near-infrared bands such as band2 (841–876 nm), blue bands such as band3 (459–479 nm), green bands such as band4 (545–565 nm) of MODIS for each date and tile were extracted, mosaicked, clipped to the extent of study area and resampled to 1000 m resolution data.

### Gaofen-1 (GF-1) data

Gaofen-1(GF-1) was launched on April 26, 2013 from the Jiuquan Satellite Launch Center. The civilian High-Definition Earth Observation Satellite (HDEOS) program was proposed in 2006 and received approval in 2010. Gaofen-1 is the first of six planned HDEOS spacecraft to be launched between 2013 and 2016. The satellite’s primary goal is to provide Near-Real-Time (NRT) observations for disaster prevention and relief, climate change monitoring, geographical mapping, environmental and resource surveying, as well as precision agriculture support. The GF-1 multispectral data have four bands with spatial resolution of 8 m and panchromatic data with spatial resolution of 2 m. Data fusion and geometric correction was carried using ERDAS software with multispectral and panchromatic data of GF-1.

### Meteorology and population data

**Figure 10:**
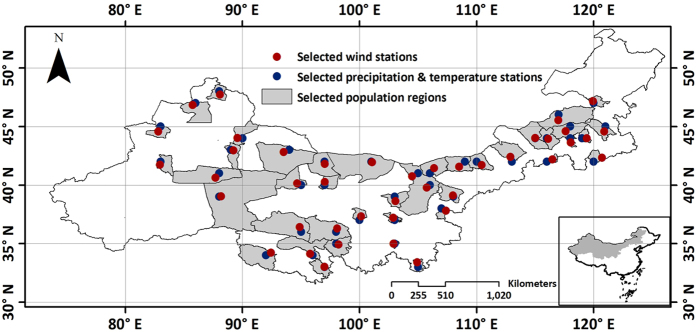
Location of temperature, precipitation, wind speed stations and population regions generated from ArcGIS 9.3 software developed by ESRI (Environmental Systems Research Institute). ArcGIS 9.3 software was downloaded from http://arcmap.software.informer.com/9.3/. It includes 42 stations of temperature, precipitation and wind speed data and 41 counties population data from 2001 to 2015 in northern China.

Temperature, precipitation and wind speed data of 42 stations and population data of 41 counties from 2001 to 2015 in northern China were collected from the National Climatic Data Center and Statistics Division of China. Mean temperature, precipitation and wind speed data of each county from 2001 to 2015 were used in this study (Fig. 10). And 33 fieldwork investigation points as interpretation key were used to perform SVM classification for GF-1 data.

### SVM classification

SVM is a range of classification and regression algorithm that has been formulated from the principles of statistical learning theory developed by Vapnik^19^. This type of classification method for remote sensing images has many advantages. The most direct advantages are that the internal structure is uniform, the boundaries between different categories are more obvious, and the classification accuracy is improved^20^,^21^,^22^. In this study, the kernel type is polynomial and the degree of kernel polynomial is two for the SVM algorithm.

### Normalized Difference Desertification Index (NDDI) calculation

In order to effectively monitor aeolian desertification distribution, band1 and band2 of MODIS were selected to calculate NDDI using the following equation^23^.


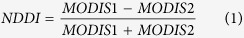


Where *NDDI* is the Normalized Difference Desertification Index; *MODIS1* is band1 of MODIS; *MODIS2* is band2 of MODIS.

### Mean Absolute Distance (MAD) calculation

Mean Absolute Distance (MAD) was calculated by the equation (2).


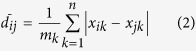


Where *i* is the line number of the pixel, *j* is the column number of the pixel, 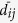 is the MAD, 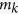 is the number of pixels in the k serie, 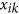 and 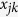 are the image values in the *k* serie, and *n* is the total number of MODIS-NDDI time series (n = 46).

### Convergent cross mapping (CCM) model

Convergent cross mapping (CCM) model is a statistical test for a cause-and-effect relationship between two time series variables that, like the Granger causality test, seeks to resolve the problem that correlation does not imply causation. While Granger causality is best suited for purely stochastic systems where the influences of the causal variables are separable (independent of each other), CCM is based on the theory of dynamical systems and can be applied to systems where causal variables have synergistic effects. The test was developed in 2012 by the lab of George Sugihara of the Scripps Institution of Oceanography, La Jolla, California, USA^24^.

## Additional Information

**How to cite this article**: Feng, L. *et al*. The dynamic monitoring of aeolian desertification land distribution and its response to climate change in northern China. *Sci. Rep.* **6**, 39563; doi: 10.1038/srep39563 (2016).

**Publisher's note:** Springer Nature remains neutral with regard to jurisdictional claims in published maps and institutional affiliations.

## Figures and Tables

**Figure 1:**
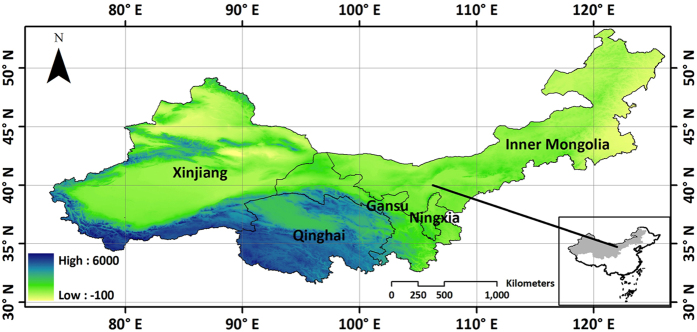
Location of study area generated from ArcGIS 9.3 software developed by ESRI (Environmental Systems Research Institute). ArcGIS 9.3 software was downloaded from http://arcmap.software.informer.com/9.3/. It includes Xinjiang Uyghur Autonomous Region, Qinghai Province, Gansu Province, Ningxia Hui Autonomous Region and Inner Mongolia Autonomous Region with eight famous deserts in China.

**Table 1:** Comparison of different fusion algorithms.

Evaluation Indexes	Fusion method	Band1	Band2	Band3	Band4
Mean	Raw multispectral image	401.69	509.26	485.89	421.18
	PCA	304.13	357.76	324.97	377.71
	Multiplicative	183197.55	233098.74	222776.04	191002.31
	Brovey Transform		163.55	155.58	137.56
Standard deviation	Raw multispectral image	61.94	95.99	102.44	53.69
	PCA	38.72	59.23	61.76	52.82
	Multiplicative	49340.83	69639.47	70646.69	44755.76
	Brovey Transform		25.89	26.74	28.55
Entropy	Raw multispectral image	6.52	6.74	6.89	6.28
	PCA	6.24	6.30	6.26	6.64
	Multiplicative	6.94	6.98	7.04	6.62
	Brovey Transform		5.89	6.50	5.89
Correlation coefficient	PCA	0.712404	0.710485	0.716641	0.933822
	Multiplicative	0.913198	0.932240	0.937543	0.833164
	Brovey Transform		0.703736	0.778586	0.651411

**Table 2:** Aolian desertification land area estimated from MODIS and investigation data.

Year	Aolian Desertification Land Area Estimation (Km^2^)	Aolian Desertification Land Area Investigation (Km^2^)	Relative Error (%)
2004	1714806	~1726700	~0.69
2009	1758942	~1706700	~3.06
2014	1724094	~1701600	~1.32
